# Obstructive Jaundice Mimicking Pancreatic Cancer: An Unusual Presentation of Autoimmune Pancreatitis

**DOI:** 10.7759/cureus.45970

**Published:** 2023-09-26

**Authors:** Tiwalade Ogunlaja, Efe Oni, Morris Ibeawuchi, Lubna Sattar, Filagot D Eshete, Felix B Agyebinti

**Affiliations:** 1 General Surgery, Peoples Friendship University of Russia, Moscow, RUS; 2 Internal Medicine, American University of Antigua, Osbourn, ATG; 3 Medicine and Surgery, University of Benin, Benin, NGA; 4 Medicine, Shadan Institute of Medical Sciences, Hyderabad, IND; 5 General Surgery, Jimma University, Jimma, ETH; 6 Internal Medicine, University of Ghana Medical Centre, Accra, GHA

**Keywords:** immunosuppressive therapy, endoscopic ultrasound, pancreatic cancer, obstructive jaundice, autoimmune pancreatitis

## Abstract

Autoimmune pancreatitis (AIP) is an uncommon variant of chronic pancreatitis characterized by inflammatory changes within the pancreatic tissue triggered by autoimmune mechanisms. It is known to mimic pancreatic cancer due to its similar clinical and radiological presentations. We underline a case of a 55-year-old male who presented with weight loss, jaundice, and pruritus. Radiological imaging suggested a pancreatic mass, raising suspicion of malignancy. However, subsequent evaluation, absence of parenchymal tissue and lymphoplasmacytic cells on endoscopic ultrasound-guided biopsy, and elevated serum immunoglobulin G4 level resulted in the diagnosis of AIP. Our case emphasizes that AIP should be included in the differential diagnosis of obstructive jaundice, especially when clinical and radiological findings are inconclusive for pancreatic cancer.

## Introduction

Autoimmune pancreatitis (AIP) is an uncommon variant of chronic pancreatitis characterized by inflammatory changes within the pancreatic tissue triggered by autoimmune mechanisms leading to dense lymphoplasmacytic infiltration with fibrosis [1]. AIP is now considered an immunoglobulin G-4 (IgG4)-related disease, which is a multisystem disease. Being an uncommon disease, the global prevalence of AIP is less than 1% per 100,000 individuals annually. AIP generally manifests in middle-aged to elderly patients and is more common in males as compared to females [2]. AIP can present various clinical manifestations, including obstructive jaundice, abdominal pain, weight loss, and pancreatic dysfunction, mimicking a presentation similar to pancreatic carcinoma [3]. One of the challenging aspects of AIP is its propensity to mimic pancreatic cancer due to overlapping clinical manifestations and radiological findings. Here, we underline a case report of obstructive jaundice caused by AIP, which initially raised suspicion for pancreatic malignancy.

## Case presentation

A 55-year-old male was brought to the emergency department with progressive jaundice for four months. He also complained of occasional pruritus, dark-colored urine, and undocumented weight loss. He had no history of abdominal pain, diarrhea, or constipation, and his medical record was unremarkable for pancreatitis, autoimmune diseases, or malignancies. He had a seven-year history of smoking when he was 30, with no history of alcohol or substance abuse.

On examination, his vitals were stable. He looked jaundiced, and an abdominal examination revealed mild epigastric tenderness without palpable masses. Initial serum and biochemical evaluations are tabulated in Table 1. Autoimmune screening involving antinuclear antibodies and anti-smooth muscle antibodies was negative.

**Table 1:** Results of hematological and biochemical parameters. ASP: aspartate aminotransferase.

Lab test	Result	Reference value
Hemoglobin	10.2 g/dL	13.4 - 17.6 g/dL
Serum lipase	139 IU/L	0-160 IU/L
Random blood sugar level	170 mg/dl	<200 mg/dl
Serum amylase	122 IU/L	0-140 IU/L
Creatinine	1.01 mg/dL	0.5 - 1.2 mg/dL
Serum alanine aminotransferase	89 IU/L	7 - 56 IU/L
Serum AST	104 IU/L	8 - 38 IU/L
Alkaline phosphatase	177 IU/L	45 - 115 IU/L
Total bilirubin	3.9 mg/dL	0.1 - 1.2 mg/dL
Serum direct bilirubin	3.9	< 0.3 mg/dl
Serum gamma-glutamyl transferase	210 IU/L	05-40 IU/L

**Figure 1:**
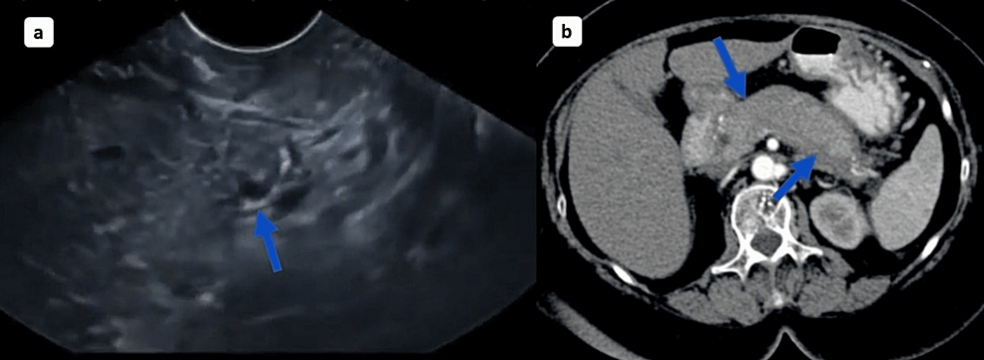
Abdominal ultrasonography reveals a dilated pancreatic duct wall (a), and CT abdomen reveals pancreatic enlargement with pancreatic head mass (b). CT: computed tomography.

Abdominal ultrasound revealed dilated common bile and pancreatic ducts, as well as diffuse pancreas enlargement (Figure 1a). Computed tomography (CT) scan of the abdominal region demonstrated a 3.5 cm pancreatic head mass with associated upstream ductal dilation (Figure 1b). These radiological findings raised concern for pancreatic cancer.

**Figure 2:**
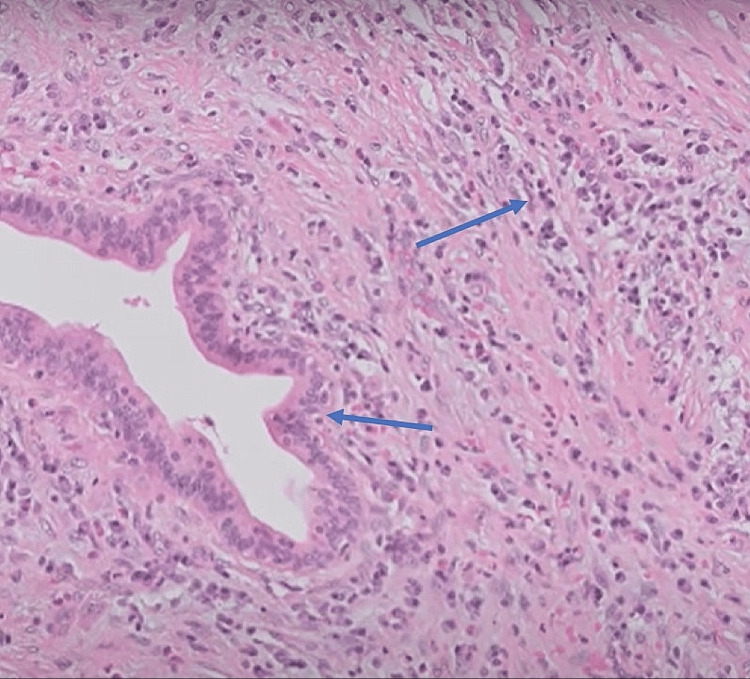
A tissue biopsy shows fibrocollagenous tissue with scattered lymphoplasmacytic infiltrates containing neutrophils and lymphocytes without parenchymal tissue.

Given the clinical presentation and radiological findings, the patient underwent endoscopic ultrasound (EUS)-guided fine-needle aspiration (FNA) of the pancreatic mass. The cytological analysis of the FNA specimen was inconclusive, showing mixed inflammatory cells but no evidence of malignancy. A biopsy of the lesion (Figure 2) showed fibrocollagenous tissue with scattered lymphoplasmacytic infiltrates containing neutrophils and lymphocytes without parenchymal tissue suggestive of AIP. Endoscopic retrograde cholangiopancreatography (ERCP) was performed with biliary stent placement to relieve the obstructive jaundice. Further evaluation revealed significantly elevated serum IgG4 levels at 201 mg/dL (normal range: <135 mg/dL) and CA-19-9 levels at 49 IU/ml (normal range: <37 IU/ml). This prompted further investigation into the possibility of autoimmune pancreatitis. A repeat EUS was performed, which revealed a diffusely enlarged and hypoechoic pancreas with irregular borders consistent with the features of AIP. A provisional diagnosis of AIP was made, and he was managed with prednisone 5 mg/day for four weeks. He was discharged on frequent follow-up, and he showed gradual improvement.

## Discussion

AIP presents diagnostic challenges necessitating a thorough evaluation of clinical manifestations, imaging results, and laboratory findings. In cases of suspected AIP, it is crucial to consider other differentials, as its clinical manifestations may overlap with those of other pancreatic diseases, including pancreatic carcinoma and diverse forms of chronic pancreatitis [4]. Among chronic pancreatitis, alcoholic pancreatitis, idiopathic pancreatitis, and hereditary pancreatitis share similar manifestations and laboratory abnormalities to those observed in AIP, which can be differentiated from AIP by the history of heavy alcohol drinking, family history of AIP, and diagnosis of exclusion [3,4]. AIP can present various clinical manifestations, including obstructive jaundice, abdominal pain, weight loss, and pancreatic dysfunction, mimicking a presentation similar to pancreatic carcinoma and chronic pancreatitis [5].

Diagnosing AIP involves a complete clinical assessment, serological tests, and imaging findings [6]. Many criteria were proposed over time to diagnose AIP [7-10]. In 2002, the Japan Pancreas Society introduced the initial diagnostic criteria for AIP, providing a fundamental benchmark for diagnosis [7]. Subsequently, the Intractable Pancreatic Disease research team expanded these diagnostic criteria. Asan Medical Center proposed the inclusion of response to steroid treatment in existing diagnostic criteria [8]. Furthermore, the criteria were expanded to include histology and cytology [9,10]. The Mayo Clinic further modified it, and the latest criteria comprehensively incorporate clinical picture, imaging, lab values, pathology, and response to steroid therapy [10]. A comparative overview of diverse formulations of diagnostic criteria for AIP is shown in Table 2. AIP is usually managed with steroid therapy, and the recommended dose for AIP is 0.4 to 0.6 mg/kg/day for four to eight weeks [11]. In cases of ineffective steroid treatment, immunomodulatory therapy is the alternative option, which may include rituximab, mycophenolate, or azathioprine. Obstructive jaundice may be relieved by biliary drainage intervention [12]. The disease prognosis is favorable, as relapse is seen only in 50% of the patients, and most of the patients recover with effective treatment [13].

**Table 2:** Criteria proposed for AIP diagnosis over time. MRCP: magnetic resonance cholangiopancreatography, LP: lymphoplasmacytic, CT: computed tomography, ERCP: endoscopic retrograde cholangiopancreatography, Ig: immunoglobulin, MRI: magnetic resonance imaging.

Parameter	Japanese Pancreatic Society [7]	Revised Korean Criteria [8]	Asian Criteria [9]	Mayo Clinic (HISORt) [10]
Serology	Gammaglobulin, IgG4, autoantibodies	IgG4, autoantibodies	IgG4, autoantibodies, IgG	IgG4
Histology	LP	LP, IgG4 cells	LP, IgG4 cells	LP, IgG4 cells
ERCP/CT/MRI	ERCP	ERCP/MRCP	ERCP	ERCP/MRCP
Steroid response	No	Yes	Yes	Yes
Extrapancreatic	Excluded	Included	Excluded	Included

Our case highlights the diagnostic challenges of autoimmune pancreatitis, especially when it presents obstructive jaundice and radiological features that mimic pancreatic cancer. The patient's initial clinical presentation of jaundice, pruritus, weight loss, and an elevated CA 19-9 level raised suspicion for malignancy. However, a high level of IgG-4, biopsy findings, and clinical improvement in using steroids confirm the diagnosis of AIP.

## Conclusions

AIP is a rare but important differential diagnosis in patients presenting with obstructive jaundice, as it can closely mimic pancreatic cancer in clinical and radiological findings. Our case highlights the significance of a thorough diagnostic workup, including ERCP and tissue biopsy, to differentiate between AIP and pancreatic malignancy and avoid unnecessary surgical interventions. Early recognition and appropriate management, often involving corticosteroid therapy, can lead to a favorable outcome, as demonstrated in our case. Clinicians should maintain a high index of suspicion for AIP in cases of obstructive jaundice, particularly when there is an atypical clinical and radiological presentation, as timely intervention can significantly improve patient outcomes and quality of life.
